# Unconscious uncoupling: dysfunctional neurovascular responses to low glucose in type 1 diabetes and impaired hypoglycemia awareness

**DOI:** 10.1172/JCI205273

**Published:** 2026-04-15

**Authors:** Stephanie A Amiel, Fernando O Zelaya

**Affiliations:** 1 Emeritus Professor of Diabetes Research, Department of Diabetes, School of Cardiovascular and Metabolic Medicine, King’s College London, United Kingdom.; 2 Honorary Consultant, King’s College Hospital NHS Foundation Trust, London, United Kingdom.; 3 Department of Neuroimaging, Institute of Psychiatry, Psychology and Neuroscience, King’s College London, United Kingdom.

## Abstract

Approximately 25% of individuals with type 1 diabetes (T1D) experience impaired awareness of hypoglycemia (IAH), a weakening of symptomatic neurohumoral responses to falling glucose levels that sharply increases risk of severe hypoglycemia. A recent study by Filip et al. used MRI-based arterial spin labeling to compare regional cerebral blood flow (CBF) responses to experimental hypoglycemia across 3 groups: individuals without T1D and individuals with T1D, with or without IAH. All groups showed a CBF response to hypoglycemia in brain regions involved in learning and interoception, among others, but the responses were qualitatively different between groups and blunted in the presence of IAH. The association between the regional CBF and the hormonal responses to hypoglycemia was inverted in IAH, compared with that in individuals with preserved awareness. The findings add to work linking changes in cognitive processing to IAH development and its persistence in some individuals.

## Central nervous system responses regulate response to hypoglycemia

Blood glucose concentrations in humans are kept within a very narrow range, despite large fluctuations in glucose availability (e.g., the fed versus fasted states) and whole-body glucose utilization (e.g., rest versus exercise). The brain, normally an obligate glucose consumer, begins to malfunction when circulating glucose falls below 54 mg/dl (3 mmol/l), and this malfunction becomes dangerous at very low glucose, involving confusion, seizure, and coma (1). Episodes of severe hypoglycemia, in which confusion renders the affected individual unable to recognize the event and undertake coordinated self treatment (1), remain a clinical problem, despite major advances in diabetes therapies and therapeutic technologies. In type 1 diabetes (T1D), with its extreme insulin deficiency, the risk of severe hypoglycemia can be reduced by using semiautomated subcutaneous insulin delivery from a small pump, driven by continuous monitoring of interstitial tissue glucose concentrations by a subcutaneous sensor. Such hybrid closed-loop systems still require user input. Up to 20% of people using such devices still report experiencing at least one severe hypoglycemia episode per year, with half that number reporting more than one episode (2).

**Figure 1:**
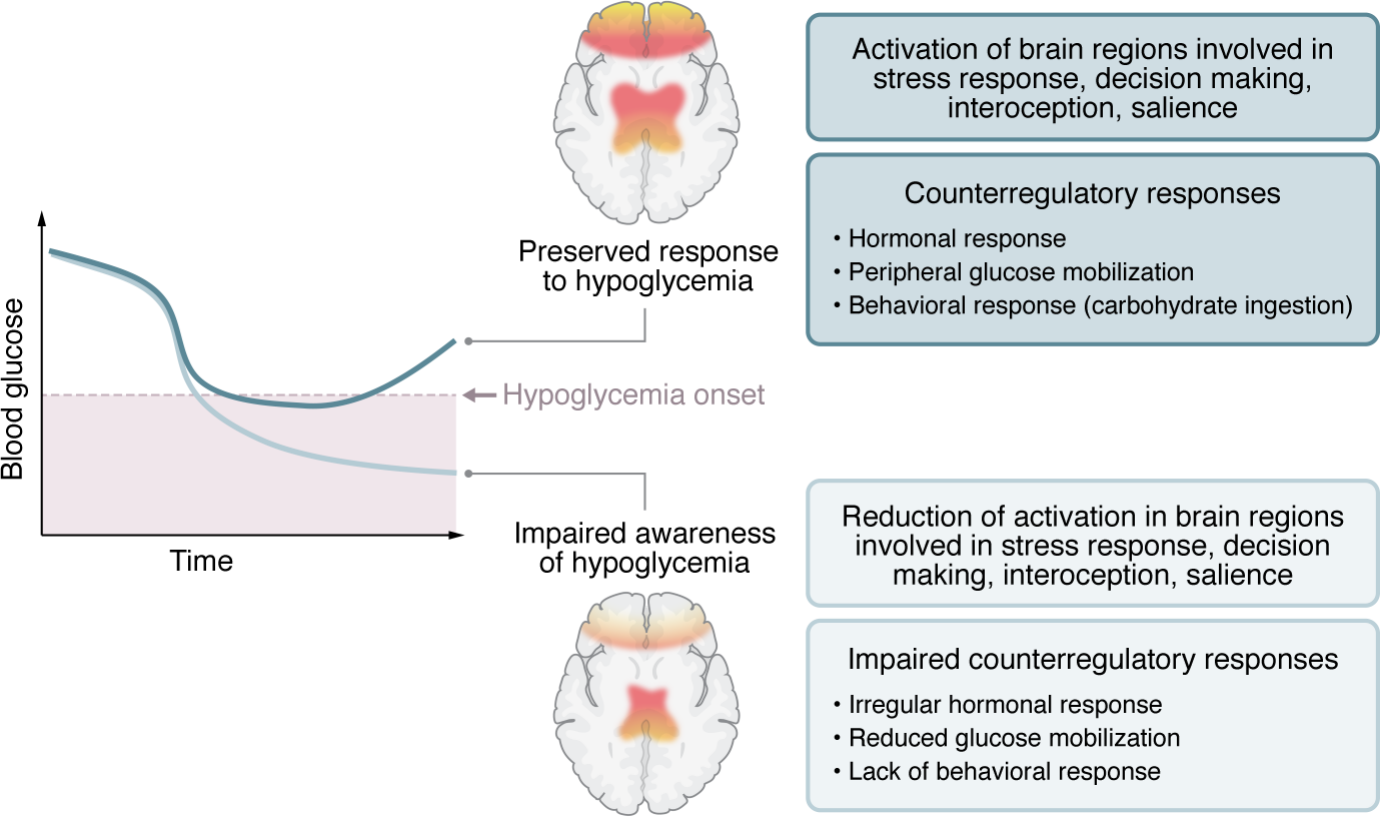
Altered CBF responses associated with IAH may underlie ineffective counterregulatory responses to falling blood glucose levels. In most individuals, hypoglycemia evokes protective responses that involve activation of and enhanced CBF to brain regions involved in decision making, interoception, and detection of salience. These brain regions coordinate counterregulatory responses to falling glucose levels that mobilize glucose stores and elicit food-seeking behavior to avoid progression to severe hypoglycemia. However, a subset of individuals with T1D experience IAH accompanied by failure to mount a counterregulatory response, raising their risk of life-threatening severe hypoglycemia. Using MRI imaging of CBF combined with experimental hypoglycemia, Filip et al. (6) observed alterations in CBF and hormonal responses to hypoglycemia in individuals with T1D and IAH that may represent progressive neurovascular dysfunction. The findings raise the question of whether intrinsic differences in neurovascular responsiveness may predispose some individuals with T1D to develop IAH despite adequate control of blood glucose.

During hypoglycemia, the brain is both the victim of falling glucose and the instigator and coordinator of protective responses that act to restore concentrations. Specific brain regions sense the reduced glucose supply and stimulate a counterregulatory neuro-humoral response that enhances glucose release and production from hepatic stores, reduces peripheral tissue glucose uptake, and increases cerebral blood flow (CBF), thereby maximizing glucose supply to the brain (3). These responses are accompanied by symptomatic stress responses, which are perceived and processed by the brain. To prevent severe hypoglycemia, counterregulatory responses must start before the onset of cognitive decline (Figure 1, top). In healthy individuals without diabetes, endogenous defences are generally effective. However, in individuals with insulin-treated diabetes, in whom the insulin stimulus is excessive and the hormonal responses weakened, neural and cognitive perception of symptoms that facilitate making the coordinated response of carbohydrate ingestion become key. About one quarter of people with T1D and perhaps one tenth of those with insulin-treated type 2 diabetes experience impaired awareness of hypoglycemia (IAH), which is associated with delayed and diminished counterregulatory responses (Figure 1, bottom). IAH greatly increases the risk of severe hypoglycemia.

## Impaired awareness of hypoglycemia persists despite therapeutic advances

Experiencing multiple episodes of hypoglycemia downregulates responses to subsequent episodes, and avoiding exposure to plasma glucose below 54 mg/dl (3.0 mmol/l) can restore awareness (shown experimentally in ref. 4, among others). Yet, around 30% of people using hybrid closed loop insulin delivery devices experience IAH, a prevalence indistinguishable from that in people using conventional insulin pumps or manual injections, despite demonstrably reduced hypoglycemic exposure in device users (2). The proportion of adults with T1D presenting with severe hypoglycemia seems to remain fixed at around 10% (2) and may be as high as 25% (5). Research exploring why some people seem particularly prone to IAH and are apparently unable to engage successfully with hypoglycemia avoidance strategies that should restore awareness has increasingly focused on the brain.

## Findings link progressive neurovascular dysfunction to IAH

In this month’s JCI, Filip and colleagues report a new neuroimaging study of regional brain blood flow and its fluctuations in hypoglycemia, studying individuals with and without type 1 diabetes (T1D) and those with and without IAH (6). They report that, in volunteers without T1D, controlled experimental hypoglycemia increased CBF in brain regions involved in learning and making behavioral responses. Importantly, experimental hypoglycemia also increased CBF in regions involved in awareness of internal state (interoception) and assessing the experience (salience). Moreover, these CBF increases colocalized with suppression of very low frequency vasomotor oscillations that are believed to reflect neurogenic and metabolic cerebral autoregulation (7); suppression of these vasomotor oscillations was also observed in brain regions involved in glucose sensing, stress responses, and memory. In participants without T1D, the rise in regional brain blood flow correlated with plasma epinephrine and cortisol responses. In contrast, participants with T1D showed less suppression of vasomotor oscillations in some brain regions, which was consistent with a degree of autonomic disruption. However, Filip et al.’s analysis of this group included those with IAH, in whom the increase in CBF was also reduced relative to healthy participants, especially in brain regions involved in learning and behavioral responses, and in interoception, salience, and decision making. In individuals with T1D and IAH, the correlation between regional CBF and hormone responses was exaggerated, but this relationship was notably inverted in individuals with T1D and preserved awareness of hypoglycemia.

Filip et al.’s study (6) demonstrates the utility of employing MRI-based arterial spin labelling (ASL) to derive quantitative and noninvasive maps of the distribution of blood flow in the brain. ASL is perhaps the only method that can be safely employed to quantitatively examine the temporal, physiological response of the brain to a challenge such as hypoglycemia. The authors acknowledge limitations. Perceived differences in brain maps between groups must be confirmed by formal statistical comparison, and correlations with hormonal markers in active regions should be defined by reference to a brain atlas. Temporal drift in ASL data occurs during scanning, and small signals attributable to physiological phenomena must be distinguished from background noise and motion artifact, an effect that might be mitigated by using ASL pulse sequences that do not employ gradient recalled echoes for acquisition of the MR signal. Despite these problems, the authors describe outcomes with clinical plausibility. They suggest that, in individuals with IAH, the altered CBF response to hypoglycemia may represent a progression from a neurovascular dysfunction that initially occurs in individuals with T1D without IAH.

## Untangling neurovascular and cognitive contributions to IAH

From a clinical perspective, the function of the affected brain regions may be key. Changes in regional brain perfusion may be both a consequence and driver of regional neuronal activation, while changes in sympathetic control of vasomotor tone, measured here in low frequency oscillations, may also reflect functional alterations in these brain regions.

In the 1980s, arteriovenous difference studies showed that brain glucose extraction was preserved during induced hypoglycemia in people with T1D and IAH (8). This observation is consistent with an upregulation of brain glucose uptake, an adaptation that might sustain intracellular glucose, and therefore brain function, during subsequent hypoglycemia. This adaptation was suggested to be responsible for delayed triggering of counter-regulatory responses. But it was evident clinically, and from experimental studies, that higher brain (i.e., cortical) function was not better maintained during hypoglycemia in IAH and more recent neuroimaging studies have not always confirmed the original finding (9, 10). Early studies of regional brain responses to hypoglycemia found changes not just in brain regions involved in glucose sensing, stress responses (including the hypothalamic-pituitary pathway), and sympathetic responses, but also in cortical and subcortical regions involved in memory, salience, aversion, appetite control, recall, and memory (11).

The present study also identified IAH-associated changes in brain networks involved in interoception and salience. By definition, IAH is a failure of perception of the body’s state, and a failure of vasomotor responsiveness in brain regions involved in perception would exaggerate any impairment in the generation of stress responses. While impaired interoception may be a feature of reversible IAH, researchers have started to investigate the possibility that reduced interoceptive ability may increase risk for developing IAH (12). Meanwhile, an inability to attribute appropriate salience to or feel aversion for a dangerous experience, such as severe hypoglycemia, constitutes a barrier to engaging in behaviors to avoid it. Ten years ago, psychologists described a subgroup of Swedish individuals with T1D who were at high risk of severe hypoglycemia yet expressed low concern about their hypoglycemia (13). In the UK, people with IAH also gave low priority to hypoglycemia avoidance and described cognitions such as drive to avoid hyperglycemia, underestimating hypoglycemia, normalizing impaired awareness, and wanting to avoid being seen as sick (14).

## Implications for IAH interventions and future studies

Filip et al.’s neuroimaging studies, together with the qualitative data, have clinical implications, particularly for people with T1D and IAH that seems resistant to evidence-based interventions to reduce severe hypoglycemia risk and improve hypoglycemia awareness. Patient-focused structured education designed to help people self adjust their insulin regimen at the time of each administration enables flexibility of lifestyle, encompassing variation in food choices, meal timing, exercise, and alcohol consumption. This education reduces severe hypoglycemia and, where measured, improves hypoglycemia awareness, together with improved quality of life (15–18). The development of continuous glucose monitoring, and latterly, hybrid closed loop insulin therapy, have further reduced hyper- and hypoglycemia incidence. Given that hypoglycemia exposure is a major risk factor for developing IAH, it remains puzzling that this technology can reduce hypoglycemia exposure without improving awareness (19, 20). It is as though the technological awareness provided by continuous glucose monitoring is not fully replicating the neural messages that physiological awareness provides. Meanwhile, as shown in clinical studies, a combination of both education and technology and resulting reductions in severe hypoglycemia and improved awareness scores was accompanied by increased CBF responses to hypoglycemia in brain regions involved in decision making and interoception — but not in cortical and thalamic networks involved in arousal and emotion processing (21). Might abnormalities of neurovascular response in the latter brain regions underlie the persistence of IAH in people using technology? It is noteworthy that IAH was defined in the Filip et al. study (6) by questionnaire and the group differences described seem independent of symptomatic and hormonal responses during the study.

In IAH, a failure of aversive response, added to inability to feel unpleasant symptoms, will diminish motivation to address the problem. In a formal trial, we investigated an intervention using motivational interviewing and cognitive behavioral theory to address unhelpful health beliefs around hypoglycemia in treatment-resistant IAH, which successfully reduced problematic hypoglycemia and improved hypoglycemia awareness, while also improving quality of life and mental health scores (22). The intervention had lasting benefit in people also using advanced diabetes technologies (23). Meanwhile, the present data leave us with a “chicken-and-egg” progression: they suggest that some people may be preprogrammed to respond to hypoglycemia exposure with IAH, perhaps through reduced interoceptive capacity (12) or reduced ability to recognize their emotions (24) but whether the observed differences in the regional brain vasomotor responses to hypoglycemia are causal or responsive is not known. This predisposition toward IAH may be mediated by intrinsic differences in patterns of brain vasomotor responsiveness that are differentially affected as a further complication of diabetic neuropathy. Studies of reversibility may help understand causality. There is a growing body of evidence implicating cognitive and emotional processing in the pathogenesis of IAH. Its persistence, despite best efforts of technology, underlines the importance of on-going investigation.

## Conflict of interest

The authors have declared that no conflict of interest exists.

## Version 1 04/15/2026

Electronic publication
